# Influencing factors of individuals’ willingness to share public health data in big data-driven healthcare: an empirical study based on China

**DOI:** 10.3389/fpubh.2026.1795026

**Published:** 2026-03-18

**Authors:** Wenjing Wang

**Affiliations:** School of Law, Huazhong University of Science and Technology, Wuhan, Hubei, China

**Keywords:** big data-enabled healthcare, influencing factors, public health data, structural equation modeling (SEM), willingness to share

## Abstract

**Background:**

Big data-enabled healthcare provides a pivotal underpinning for the precision and efficiency of public health governance, with the effective sharing of personal public health data serving as its core prerequisite. This study aims to explore the influencing factors and action mechanisms of individuals’ willingness to share personal public health data in the context of big data-enabled healthcare, thereby providing empirical evidence for breaking the predicament of data sharing and improving the public health data governance system.

**Methods:**

A multi-stage convenience sampling method was adopted in this study. Questionnaires were distributed to populations nationwide who have used or understood big data-enabled healthcare platforms, and a total of 616 valid samples were recovered. On the basis of ensuring the questionnaire met the criteria of reliability and validity, correlation analysis and structural equation modeling (SEM) were employed to test the path relationships among variables.

**Results:**

The results indicated that the overall scale exhibited good reliability (Cronbach’s *α* = 0.897) and acceptable validity (cumulative variance explanation rate = 76.47%), with the SEM showing a good fit (*χ*^2^/df = 2.153). Perceived technological usefulness had a significantly positive impact on perceived usefulness (*β* = 0.407, *p* < 0.001). Perceived usefulness and disease severity exerted positive effects on the attitude toward data sharing (*β* = 0.311, 0.364, both *p* < 0.001), while information sensitivity had a negative effect on such attitude (*β* = −0.211, *p* < 0.001). Attitude toward sharing, legal protection of public health and medical data, and perceived behavioral control were significant positive predictors of the willingness to share data (*β* = 0.379, 0.312, 0.308, all *p* < 0.001). Moreover, the willingness to share data and the legal protection of public health and medical data jointly promoted the construction of public health data platforms (*β* = 0.404, 0.421, both *p* < 0.001).

**Conclusion:**

This study uncovers the complex action paths of multi-dimensional factors on individuals’ willingness to share personal public health data, which provides theoretical support and practical guidance for optimizing the design of big data-enabled healthcare services, strengthening the legal protection of public health data, and improving the efficiency of data sharing.

## 1 Introduction

Amid the in-depth integration of digital technologies and the healthcare sector, big data-enabled healthcare has emerged as a core engine driving the modernization of public health governance systems and enhancing the precision of public health services (1). As the core production factor of big data-enabled healthcare, personal public health data encompasses multi-dimensional content such as health monitoring records, disease diagnosis and treatment information, and physical examination reports (2). Its efficient sharing can provide solid data support for major infectious disease prevention and control, precise chronic disease management, and public health policy formulation (3), serving as a key prerequisite for realizing the “prevention-first” public health strategy.

In recent years, countries around the world have accelerated the construction of public health data sharing systems. China has also successively issued policy documents such as the Healthy China 2030 Planning Outline (4) and the Measures for the Administration of Standards, Security, and Services of National Health Medical Big Data (5), which clearly propose promoting the opening and sharing of public health data and building an integrated big data-enabled healthcare service system. Local governments in China have shown differentiated progress in policy implementation: developed provinces such as Zhejiang and Guangdong have issued provincial-level medical big data management measures and built regional health data platforms to realize interconnection of partial medical data, while central and western provinces lack specific implementation rules and have slow progress in platform construction. However, in practice, the sharing of personal public health data in China is confronted with the practical dilemma of “insufficient willingness” and “data isolation.” Although the public value of data sharing has reached a consensus, factors such as the public’s concern about data privacy leakage, perceptual bias towards the actual value of data sharing, and insufficient trust in legal protection mechanisms (6) have led to generally low participation in personal public health data sharing. A large amount of valuable data resources remain idle (7), severely restricting the large-scale application of big data-enabled healthcare in the public health field.

Particularly in the context of big data-enabled healthcare, data sharing involves multiple subjects and links, with multi-dimensional factors such as technical characteristics, individual psychology, and institutional guarantees interweaving (8), which further exacerbates the complexity of data sharing willingness. Therefore, systematically clarifying the influencing factors of individuals’ willingness to share personal public health data in the context of big data-enabled healthcare and revealing the mechanism of action among these factors have become an urgent need to break the predicament of data sharing and unlock data value.

The theoretical contributions and innovations of this study are mainly reflected in the following aspects: First, based on the Technology Acceptance Model (TAM) and the Theory of Planned Behavior (TPB), this study integrates three dimensions—technical characteristics, individual psychology, and institutional guarantees—to construct a multi-factor integrated model of personal public health data sharing willingness. Relevant studies have confirmed that the integrated model of TAM and TPB has higher explanatory power for users’ technology acceptance and behavioral willingness than a single model, and can make up for the deficiency that TAM ignores the influence of individual behavioral control and institutional factors (9). This enriches the theoretical research framework in the field of medical data sharing and makes up for the limitation of existing studies that mostly adopt single-dimensional analysis. This enriches the theoretical research framework in the field of medical data sharing and makes up for the limitation of existing studies that mostly adopt single-dimensional analysis. Second, it empirically tests the roles of variables such as perceived technological usefulness and legal protection of public health and medical data, revealing the complete action chain of “technical cognition—psychological attitude—behavioral willingness—platform construction” and deepening the understanding of the formation mechanism of data sharing willingness. Third, it expands the application scenarios of the TAM model and TPB theory in the public health field, providing theoretical reference and methodological guidance for subsequent related research.

At the practical level, this study also has important guiding value. For big data-enabled healthcare service providers, the research conclusions can provide precise directions for platform function optimization and service design upgrading, helping them improve users’ perception and trust in the value of data sharing. For relevant government departments, the research findings can offer empirical evidence for improving laws and regulations on public health data protection and formulating data governance policies, contributing to the construction of a safe, credible, and efficient institutional environment for data sharing. For public health service institutions, the research results can provide practical guidance for promoting the construction of public health data platforms and improving data sharing efficiency, facilitating the better application of big data-enabled healthcare technologies in public health governance and ultimately realizing the improvement of national health literacy.

The core content and steps of this study are as follows: First, through literature review and theoretical analysis, the connotations and correlations of core variables—including perceived technological usefulness, perceived usefulness, disease severity, information sensitivity, attitude, perceived behavioral control, legal protection of public health and medical data, willingness to share, and construction of public health medical data platforms—are clarified. After identifying research gaps, a theoretical model is constructed and research hypotheses are proposed. Second, a questionnaire is designed and a pre-survey is conducted; after optimizing the questionnaire items, a formal survey is carried out to collect valid sample data. Third, reliability and validity tests as well as correlation analysis are performed on the data, a structural equation model (SEM) is constructed, and model fitting and path testing are conducted using the maximum likelihood method. Finally, the empirical results are interpreted to reveal the influencing mechanism of each variable on sharing willingness, targeted optimization strategies are proposed, and the research limitations and future research directions are summarized.

## 2 Literature review and research hypotheses

### 2.1 Literature review

#### 2.1.1 Big data-enabled healthcare and public health data sharing

Big data-enabled healthcare relies on digital technologies such as big data and artificial intelligence to realize the integrated analysis, precise prediction, and efficient utilization of healthcare data, and has become a core underpinning for advancing the modernization of public health governance. As the core production factor of big data-enabled healthcare, personal public health data covers various types of information including health monitoring data, disease diagnosis and treatment records, and physical examination reports. Its cross-institutional and cross-scenario sharing can provide precise data support for disease early warning, chronic disease management, and public health policy formulation (10), and exhibits irreplaceable value especially in fields such as major infectious disease prevention and control and national health literacy improvement.

In recent years, countries around the world have been promoting the construction of public health data sharing systems. European and American countries have established relatively sound data sharing and privacy protection mechanisms by formulating laws and regulations such as the Health Insurance Portability and Accountability Act (HIPAA) and the General Data Protection Regulation (GDPR), providing institutional guarantees for the compliant flow of data.

China has also successively issued a series of policies and laws to promote the development of big data-enabled healthcare and the sharing of public health data, including the Basic Medical and Health Care and Health Promotion Law (11) and the Personal Information Protection Law (PIPL) (12). The PIPL clearly defines health information as “sensitive personal information” and stipulates that separate consent of individuals is required for the collection and use of health data, providing a legal basis for the protection and sharing of public health data. Up to 2024, the National Health Commission of China has built 10 national health medical big data centers and approved 15 provincial-level health medical big data pilot zones, including Zhejiang, Guangdong and Shanghai. At the provincial level, 15 provinces including Zhejiang and Shanghai have built provincial regional health medical big data platforms, realizing the interconnection and sharing of residents’ electronic health records and electronic medical records in varying degrees. In terms of technical application, big data-enabled healthcare technologies in China are mainly applied in major infectious disease early warning, chronic disease management, precise medical treatment and public health policy formulation. For example, during the COVID-19 epidemic, the national health code platform realized cross-regional epidemic prevention and control by virtue of big data technology, which verified the huge application potential of big data-enabled healthcare in the public health field (13). However, in practice, the development of big data-enabled healthcare in China still faces many problems: the digital level of primary medical and health institutions is low, the popularization of big data technology is insufficient, and the public’s privacy concerns about health data sharing are prominent (14). The problem of insufficient willingness to share personal public health data is prevalent, and factors such as concerns about privacy leakage risks, perceptual deviations in the value of data sharing, and insufficient trust in legal protection have restricted the large-scale advancement of data sharing, becoming a key bottleneck for the practical application of big data-enabled healthcare.

#### 2.1.2 Previous research on health data sharing

Existing studies on health data sharing willingness have achieved rich results worldwide, and the research perspectives are mainly focused on technical acceptance, individual psychology and institutional guarantee. From the perspective of international research, most studies focus on exploring the influencing factors of health data sharing willingness based on a single theoretical model, and verify the applicability of classic theories such as TAM and TPB in the field of health data sharing. For example, Olsen et al. (15) found that attitude and perceived behavioral control are the core factors affecting sharing willingness; Helou et al. (16) took the information sensitivity and data usefulness as the core variables to explore the sharing willingness, and confirmed the negative regulatory effect of information sensitivity.

From the perspective of domestic research in China, the research on health data sharing willingness started relatively late, and the research content is mainly focused on policy suggestions, platform construction and empirical analysis of specific groups. For example, Peng et al. (17) studied the construction mode of regional health data platforms in China and put forward targeted suggestions for breaking “data islands”; Cheng et al. (18) analyzed the medical big data acceptance of the older population in China based on the TAM-TPB model and found that perceived ease of use is the key factor affecting their acceptance.

In general, existing studies have laid a solid foundation for the research on health data sharing willingness, but there are still deficiencies in the integration of multi-dimensional factors and the construction of a comprehensive theoretical model, which provides a research space for this study to integrate TAM and TPB models and construct a multi-dimensional integrated model of technical characteristics-individual psychology-institutional guarantee.

#### 2.1.3 Research on influencing factors of healthcare data sharing willingness

##### 2.1.3.1 Factors related to technical characteristics

The Technology Acceptance Model (TAM) was proposed in 1989. Its core viewpoint is that perceived usefulness affects users’ attitudes (19), which in turn influence technology adoption willingness and behavior. This model has been widely applied in fields such as healthcare information technology evaluation and data sharing. Perceived usefulness refers to the degree to which users believe that using a certain technology can improve work or task efficiency. Some scholars have found that when users perceive that healthcare data sharing technology can effectively meet their health management needs, they are more inclined to participate in data sharing (20). Relevant studies have also verified this conclusion, pointing out that the functional practicality of big data-enabled healthcare platforms can positively enhance users’ sharing willingness (16). As a reflection of the innovation and value of the technology itself, perceived technological usefulness indirectly affects data sharing willingness by influencing users’ technical cognition, serving as an important supplementary variable in the technical characteristics dimension, and together with perceived usefulness constitutes the core influencing factors at the technical level.

##### 2.1.3.2 Factors related to individual psychological characteristics

Individual psychological cognition is a core dimension affecting individuals’ willingness to share personal public health data. Information sensitivity, as a key variable measuring the degree of individuals’ attention to privacy protection, has been proven to be negatively correlated with data sharing willingness—the higher the users’ sensitivity to personal information, the more concerned they are about privacy leakage risks in the data sharing process, thereby reducing their sharing willingness (21). Disease severity, i.e., individuals’ judgment of their own health status, also affects sharing attitude and willingness. Groups with poor health status often have higher demands for precise medical services and have a stronger recognition of the value of data sharing for disease diagnosis and treatment (22), thus showing relatively higher sharing willingness.

The Theory of Planned Behavior (TPB) provides an important theoretical framework for explaining individuals’ behavioral willingness and behavior (23). The theory points out that attitude, subjective norm, and perceived behavioral control jointly determine individuals’ behavioral willingness, and behavioral willingness further predicts actual behavior (24). In summary, attitude and perceived behavioral control are direct factors affecting behavioral willingness. In the field of healthcare data sharing, a positive sharing attitude can significantly enhance users’ participation willingness. Perceived behavioral control, i.e., individuals’ control ability over the data sharing process and confidence in risk response, positively affects sharing willingness by enhancing users’ sense of security (25). In addition, individual characteristics such as age, education level, and occupation also exert differentiated impacts on sharing willingness. Highly educated, young and middle-aged groups usually have higher sharing willingness due to their higher acceptance of digital technologies and stronger health management awareness (26).

#### 2.1.4 Factors related to institutions

The legal protection mechanism for public health data, as a core variable at the institutional level, is a key guarantee affecting users’ data sharing willingness. In the context of big data-enabled healthcare, personal public health data involves sensitive information such as health privacy and diagnosis and treatment records, and users’ trust in data sharing is highly dependent on a sound legal system. Improved laws and regulations can clearly define the boundaries of data collection, storage, transmission, and use, clarify the rights and responsibilities of all subjects in data processing, and establish accountability and compensation mechanisms after privacy leakage, reducing data sharing risks at the institutional level. A study by Kühnel and Wilke (27) confirmed that when there is a sound national-level data protection law with strong enforcement, users’ concerns about data sharing are significantly reduced, and their participation willingness is notably improved. Research by Mayumi and Kaori (28) also showed that the higher users’ awareness of policies and the stronger their trust in legal protection of data security, the more willing they are to take the initiative to share personal public health data. The essence of this trust is that the legal system provides a “risk safety net” for data sharing, enabling users to perceive that their rights and interests can be effectively protected, thereby lowering the psychological threshold for participating in data sharing. At present, China has established a legal system for health data protection with the PIPL as its core. This law clarifies the special protection rules for health information as “sensitive personal information,” requiring data processors to obtain separate consent from individuals and adopt strict safety protection measures. The perception of “legal protection of medical data” in this study is based on the public’s perception of this regulatory system.

#### 2.1.5 Deficiencies of existing research

Existing studies have explored the influencing factors of healthcare data sharing willingness from multiple dimensions such as technology, individuals, and institutions, but there are still the following deficiencies: First, most studies focus on a single dimension or a few variables, lacking integrated analysis of multiple dimensions including technical characteristics (perceived technological usefulness, perceived usefulness), individual psychology (disease severity, information sensitivity, attitude, perceived behavioral control), and institutional guarantees (legal protection of public health and medical data), making it difficult to fully reveal the formation mechanism of data sharing willingness. Second, some studies have not clarified the action paths between variables, and insufficiently explored the intermediate mechanisms of “how technology affects attitude through cognition and how attitude combines with institutional factors to act on willingness.” Third, empirical research on public health data sharing in the context of big data-enabled healthcare is relatively limited, lacking precise verification of the effect intensity and paths of variables such as legal protection and perceived technological usefulness, and failing to fully focus on the subsequent impacts of various variables on the construction of public health data platforms.

Based on this, this study strengthens the correlation analysis between perceived technological usefulness and perceived usefulness on the basis of the TAM model; draws on the core logic of the TPB theory to take attitude and perceived behavioral control as direct variables affecting sharing willingness; incorporates the institutional variable of legal protection of public health and medical data in combination with the scenario characteristics of public health data sharing, and at the same time includes individual psychological variables such as disease severity and information sensitivity. A multi-dimensional integrated model of “technical characteristics, individual psychology, institutional guarantees, sharing willingness, and platform construction” is constructed to empirically test the action paths between variables, filling the gaps in existing research.

### 2.2 Research hypotheses

Based on the aforementioned literature review and theoretical foundation, combined with the characteristics of public health data sharing in the context of big data-enabled healthcare, this study constructs a theoretical framework of “technical characteristics—individual psychology—institutional guarantees—sharing willingness—platform construction” and proposes 11 research hypotheses.

#### 2.2.1 The relationship between technical characteristics and perceived usefulness

Perceived technological usefulness is a direct reflection of the innovation, practicality, and value potential of the technology itself, reflecting users’ objective cognition of the ability of big data-enabled healthcare technologies to solve health management and public health governance problems (29). In the context of big data-enabled healthcare, the core value of technology lies in realizing precise disease prediction, dynamic health monitoring, and efficient public health decision-making through integrated data analysis, and data sharing is a prerequisite for the technology to exert its role. According to the core logic of the TAM model, users’ cognition of the technology’s own value directly affects their judgment of the technology’s application value. When users perceive that big data-enabled healthcare technologies can effectively improve disease prevention and control efficiency and optimize health management services (i.e., high perceived technological usefulness), they will naturally form the idea that “data sharing is a key link to realize these technological values” (30), thereby recognizing the practical significance and utility value of data sharing, i.e., improving perceived usefulness. Accordingly, the following hypothesis is proposed:

*H1*: Perceived technological usefulness has a significantly positive impact on perceived usefulness.

#### 2.2.2 Subsequent impacts of perceived usefulness

As the core subjective cognition of users on the value of data sharing behavior, perceived usefulness serves as a key bridge connecting technical characteristics with individual behavioral attitudes and institutional trust. From the perspective of the formation logic of behavioral attitudes, an individual’s attitude towards a certain behavior is essentially a subjective trade-off between the “benefits and costs” of the behavior. When users clearly perceive that data sharing can bring them practical benefits such as precise health assessment and personalized medical advice (i.e., high perceived usefulness), they will form a judgment that “the benefits of data sharing outweigh the costs,” thereby fostering a positive attitude towards sharing (31). From the perspective of institutional cognition correlation, when users highly recognize the practical value of data sharing, they will pay more attention to how to ensure the safety and standardization of this “valuable behavior” (32). Therefore, the improved legal protection mechanism for public health and medical data, as a core institutional arrangement to reduce data sharing risks and protect user rights and interests, will become the focus of users’ attention, and their depth of cognition and trust in the legal protection system will also increase accordingly. Accordingly, the following hypotheses are proposed:

*H2*: Perceived usefulness has a significantly positive impact on the attitude towards personal public health data sharing.

*H3*: Perceived usefulness has a significantly positive impact on the cognition of legal protection of public health and medical data.

#### 2.2.3 Impacts of individual psychological characteristics

Individual psychological characteristics are core endogenous factors affecting data sharing attitudes and perceived behavioral control, among which disease severity and information sensitivity act on user decisions from two opposite dimensions: benefit expectation and risk concern.

In terms of disease severity, individuals’ judgment of their own health status directly determines the intensity of their demand for precise medical services. Groups with poor health status, chronic diseases, or a history of serious illnesses often face higher health risks and medical needs, and the accuracy of big data-enabled healthcare is highly dependent on the integrated analysis of multi-source data (33). Data sharing can bring such groups core benefits such as more targeted diagnosis and treatment plans and disease monitoring and early warning, making their recognition of the value of data sharing significantly higher than that of healthy groups. This strong value recognition will be directly transformed into a positive attitude towards sharing, that is, the higher the perceived disease severity of an individual, the more inclined they are to recognize the necessity of data sharing and the more positive their attitude towards sharing. Accordingly, the following hypothesis is proposed:

*H4*: Disease severity has a significantly positive impact on the attitude towards personal public health data sharing.

In terms of information sensitivity, its core is individuals’ attention to personal privacy and risk aversion tendency (14). Users who are sensitive to personal information regard public health data such as physical examination reports and medical records as core privacy assets, and have strong concerns about leakage and abuse risks in the data sharing process. On the one hand, such concerns will directly affect their subjective attitude towards data sharing, believing that data sharing may lead to privacy leakage, thereby forming a negative and cautious attitude towards sharing. On the other hand, users with high information sensitivity often pay excessive attention to the potential risks of data sharing; even if they have a certain ability to respond to risks, they will subjectively weaken their sense of control over the data sharing process, believing that it is difficult to completely avoid privacy leakage risks, thereby reducing the level of perceived behavioral control. Accordingly, the following hypotheses are proposed:

*H5*: Information sensitivity has a significantly negative impact on perceived behavioral control.

*H6*: Information sensitivity has a significantly negative impact on the attitude towards personal public health data sharing.

#### 2.2.4 Direct influencing factors of sharing willingness

According to the TPB theory, attitude and perceived behavioral control are core direct factors affecting individuals’ behavioral willingness. In the scenario of big data-enabled healthcare involving sensitive information sharing, the cognition of legal protection at the institutional level, as the basis of trust, also becomes a key variable affecting sharing willingness (34).

Attitude, as an individual’s overall evaluation and emotional tendency towards data sharing behavior, directly reflects the intensity of users’ subjective willingness to participate in data sharing. A positive attitude towards sharing means that users cognitively recognize the value of data sharing and emotionally accept data sharing behavior; this positive subjective tendency will be directly transformed into the willingness to participate in data sharing. Conversely, a negative attitude towards sharing will inhibit sharing willingness. In the field of public health data sharing, the core role of attitude is reflected in “eliminating psychological resistance.” Specifically, when users hold a positive attitude towards data sharing, they will take the initiative to ignore or weaken potential risks and be more inclined to take participation behaviors. Accordingly, the following hypothesis is proposed:

*H7*: The attitude towards personal public health data sharing has a significantly positive impact on sharing willingness.

The cognition of legal protection of public health and medical data is a reflection of users’ trust in institutional guarantees and a core support for reducing concerns about data sharing risks (35). An improved legal protection mechanism can clearly define the boundaries of data collection, storage, transmission, and use, clarify the rights and responsibilities of all parties in data processing, and establish accountability and compensation mechanisms after privacy leakage, providing a “safety net” for user rights and interests at the institutional level. When users understand and trust that existing laws can effectively protect their health privacy and regulate data use behaviors, their perceived risk of data sharing will be significantly reduced, and their confidence and willingness to participate in data sharing will increase accordingly. For example, when users know that they can obtain compensation through legal channels after data leakage, they will be more willing to participate in data sharing. Accordingly, the following hypothesis is proposed:

*H8*: The cognition of legal protection of public health and medical data has a significantly positive impact on sharing willingness.

Perceived behavioral control reflects individuals’ control ability over the data sharing process and confidence in risk response, and is a key variable affecting behavioral willingness (36). When users believe that they can independently decide the scope and method of data sharing, possess the knowledge and ability to deal with issues related to data sharing, and can effectively reduce potential risks, the psychological threshold for participating in data sharing will be significantly lowered. This sense of control can alleviate users’ anxiety, enhance their sense of security in participating in data sharing, and thereby positively affect sharing willingness. Conversely, if users believe that the data sharing process is beyond their control and they cannot cope with potential risks, they will refuse to participate due to lack of security. Accordingly, the following hypothesis is proposed:

*H9*: Perceived behavioral control has a significantly positive impact on sharing willingness.

#### 2.2.5 The relationship between sharing willingness and platform construction

As the core carrier of data sharing, the construction and promotion of public health and medical data platforms rely on users’ participation willingness and institutional trust (37). Sharing willingness reflects users’ subjective tendency to participate in data sharing, and is the basis for platforms to obtain data resources and optimize functions. The stronger the users’ sharing willingness, the more willing they are to provide their own public health data to the platform, and the more willing they are to put forward suggestions for improving platform functions, providing direct resource support and demand feedback for platform construction, and thereby promoting the iterative upgrading and large-scale development of the platform. Accordingly, the following hypothesis is proposed:

*H10*: Sharing willingness has a significantly positive impact on the supportive attitude towards the establishment of public health and medical data platforms.

The cognition of legal protection of public health and medical data, as the core of institutional trust, can enhance users’ trust and recognition of the platform (38). An improved legal protection mechanism defines the compliance boundaries for the platform’s data processing behaviors and ensures the safety and standardization of data circulation on the platform. When users trust that laws can effectively restrict the platform’s data processing behaviors and protect their privacy, they will have a higher evaluation of the platform’s reliability and thus be more willing to support platform construction. This institutional trust can reduce users’ resistance to the platform, improve their enthusiasm for participating in platform construction, and lay a solid trust foundation for the promotion and operation of the platform. Accordingly, the following hypothesis is proposed:

*H11*: The cognition of legal protection of public health and medical data has a significantly positive impact on the supportive attitude towards the establishment of public health and medical data platforms.

**Figure 1:**
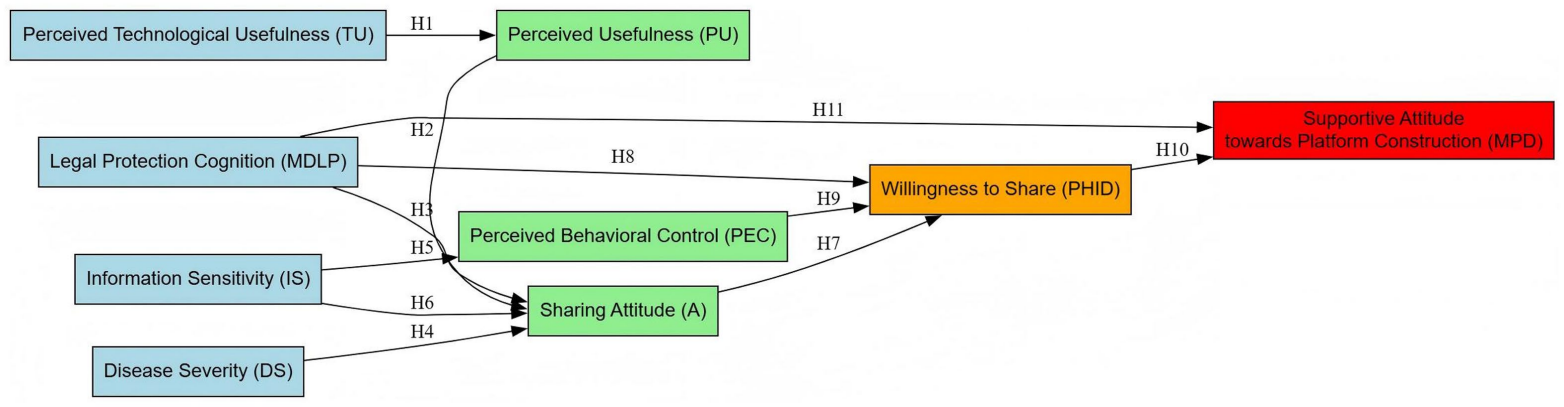
Theoretical framework of this study.

The framework diagram of this study is presented in Figure 1. This framework is based on the integration of TAM and TPB models, incorporating technical characteristics, individual psychology and institutional guarantee three dimensions. The arrows represent the hypothetical causal relationship between variables, and the numbers in brackets represent the corresponding research hypotheses (H1–H11).

## 3 Research design

### 3.1 Research participants

A mixed online and offline questionnaire survey was administered to enhance the representativeness and coverage of the research sample. Electronic questionnaires were distributed via mainstream social platforms including WeChat Official Accounts, Zhihu, and Sina Weibo (500 questionnaires issued in total); paper questionnaires were handed out to individuals with experience in using big data-enabled healthcare platforms or demands for personal health management at such venues as hospital outpatient departments, community health service centers, universities, and enterprises (200 questionnaires issued in total). During the sampling process, full consideration was given to the diversity of demographic characteristics including gender, age, occupation, and educational level to ensure the comprehensiveness of the sample. Electronic questionnaires were distributed via mainstream social platforms including WeChat Official Accounts, Zhihu, and Sina Weibo; paper questionnaires were handed out to individuals with experience in using big data-enabled healthcare platforms or demands for personal health management at such venues as hospital outpatient departments, community health service centers, universities, and enterprises. During the sampling process, full consideration was given to the diversity of demographic characteristics including gender, age, occupation, and educational level to ensure the comprehensiveness of the sample.

The sample size was determined in accordance with the sample size estimation criteria for Structural Equation Modeling (SEM). Combined with the design features of the research scale, which consists of 31 items and 9 latent variables, the principle of “number of items × 10 ~ 20” was followed (39, 40). Meanwhile, an anticipated invalid sample rate of 10% ~ 15% during questionnaire collection was taken into account, leading to the determination of a minimum sample size of 400. A total of 700 questionnaires were distributed, with 658 recovered. After rigorous screening, 42 invalid questionnaires were excluded due to excessively short response time, contradictory answers, and missing key information. Ultimately, 616 valid questionnaires were obtained, with an effective recovery rate of 88.00%. Among them, 528 online questionnaires were recovered, 465 of which were valid (effective recovery rate: 93.00%); 130 offline questionnaires were recovered, 151 of which were valid (effective recovery rate: 75.50%). The valid online samples accounted for 75.49% of the total valid samples, and the valid offline samples accounted for 24.51%, which ensured the diversity and representativeness of the sample sources.

As presented in Table 1, the demographic characteristics of the valid sample exhibit extensive coverage, scientific representativeness and rational distribution, as detailed below:

(1) Gender distribution: 345 males (56.01%) and 271 females (43.99%), showing a balanced gender ratio;
(2) Age distribution: 344 respondents (55.84%) aged 19–29, 156 (25.32%) aged 29–39, 84 (13.64%) aged 39–49, and 32 (5.19%) aged 49–60, covering the core young and middle-aged groups as well as a subset of the middle-aged and older population;
(3) Educational level: 62 respondents (10.06%) with education below bachelor’s degree, 342 (55.52%) with a bachelor’s degree, and 212 (34.42%) with a master’s degree or above. Respondents with a bachelor’s degree or higher account for 89.94% of the total sample, which is consistent with the educational distribution characteristics of groups with cognitive awareness of big data-enabled healthcare;
(4) Occupational background: 128 state functionaries (20.78%), 69 enterprise managers (11.20%), 139 general enterprise employees (22.56%), 38 freelancers (6.17%), and 200 students (32.47%), covering various occupational types with the student group accounting for the largest proportion;
(5) Big data-related work experience: 70 respondents (11.36%) engaged in big data-related work and 546 (88.64%) without such experience. The sample is dominated by ordinary users, which is highly consistent with the research objective of exploring the public’s willingness to share personal public health data.

**Table 1:** Sample demographic characteristics.

Name	Option	Frequency	Percentage (%)	Cumulative percentage (%)
Age	19–29 years	344	55.84	55.84
	29–39 years	156	25.32	81.17
	39–49 years	84	13.64	94.81
	49–60 years	32	5.19	100.00
Gender	Male	345	56.01	56.01
	Female	271	43.99	100.00
Educational attainment	Below Bachelor’s degree	62	10.06	10.06
	Bachelor’s degree	342	55.52	65.58
	Master’s degree or above	212	34.42	100.00
Employment type	Public employee	128	20.78	20.78
	Enterprise manager	69	11.20	31.98
	General enterprise staff	139	22.56	54.55
	Freelancer	38	6.17	60.71
	Student	200	32.47	93.18
	Other	42	6.82	100.00
Whether engaged in big data work	Yes	70	11.36	11.36
	No	546	88.64	100.00
Total		616	100.0	100.0

### 3.2 Questionnaire design

The questionnaire development followed a rigorous process of “literature review → theoretical grounding → item design → pre-survey optimization.” First, relevant domestic and international research findings on healthcare data sharing willingness, the Technology Acceptance Model (TAM), and the Theory of Planned Behavior (TPB) were systematically collated, and the core dimensions and item design logic of mature scales in existing studies were referenced and adapted. Second, the core dimensions of the research scale were determined by integrating the technical characteristics of big data-enabled healthcare, the sensitivity of public health data, and the policy context of public health data governance in China. Finally, the rationality, scientificity, and clarity of expression of the questionnaire items were evaluated through expert review, leading to the formulation of the initial questionnaire, as detailed in Table 2.

**Table 2:** Questionnaire content.

Variables	Statement
Perceived usefulness (PU)	PU1: Sharing personal public health data can help me obtain more accurate health assessments. PU2: Participating in data sharing can meet my need to know my health status anytime and anywhere. PU3: Data sharing provides practical assistance for my disease diagnosis/treatment or health management.
Technological usefulness (TU)	TU1: Big data medical technology can effectively improve the efficiency of disease prediction and prevention/control. TU2: Big data medical technology provides important support for public health governance. TU3: The innovation of big data medical technology is commendable and can genuinely assist health management.
Disease severity (DS)	DS1: I often suffer from illness, and my health status requires close attention. DS2: I have chronic diseases or a history of serious illness and thus have a higher demand for medical services. DS3: I believe my health condition needs improvement through precision medicine approaches.
Information sensitivity (IS)	IS1: I consider personal medical records, physical examination reports, and other public health data to be highly sensitive information. IS2: I am very concerned that sharing public health data may lead to privacy breaches. IS3: I will exercise extra caution when sharing personal health-related data. IS4: Health information shared by others also seems to involve privacy and should not be disseminated casually.
Perceived behavioral control (PEC)	PEC1: I can independently control the scope of data I share on big data medical platforms. PEC2: If I encounter problems related to data sharing, I am capable of addressing them or seeking help. PEC3: I possess the necessary knowledge to reduce potential risks associated with data sharing.
Medical data legal protection (MDLP)	MDLP1: I am aware of the relevant laws and regulations in China concerning public health data protection. MDLP2: Existing laws can clearly define the boundaries for the collection and use of public health data. MDLP3: If health data are leaked, existing laws can safeguard my rights and interests. MDLP4: Relevant departments have sufficient enforcement strength for public health data protection. MDLP5: Laws can clarify the responsibilities of data processors and reduce the risk of unauthorized use.
Attitude (A)	A1: I believe sharing personal public health data is beneficial to both myself and society. A2: I hold a positive attitude toward participating in data sharing on big data medical platforms. A3: Overall, the value of data sharing outweighs its potential risks.
Willingness to share health information (PHID)	PHID1: I am willing to share my physical examination reports, medical records, and other information with big data medical platforms PHID2: I am willing to share my personal health monitoring data (e.g., step count, blood pressure) with medical service providers. PHID3: I have no objection to sharing my public health data with doctors or other users in need. PHID4: If given the opportunity in the future, I will actively participate in public health data sharing projects.
Medical platform development (MPD)	MPD1: I support the establishment of a unified public health and medical data sharing platform. MPD2: I am willing to provide suggestions for optimizing the functions of the public health data platform. MPD3: I believe a unified data platform can make data sharing safer and more efficient.

### 3.3 Questionnaire quality assessment

#### 3.3.1 Reliability analysis

Cronbach’s Alpha coefficient was adopted to measure the internal consistency reliability of the questionnaire. In Cronbach’s Alpha assessment, a higher coefficient value indicates a higher level of internal consistency of the scale. The generally accepted evaluation criteria are as follows: a coefficient greater than 0.9 denotes excellent internal consistency; a coefficient between 0.8 and 0.9 indicates good internal consistency; a coefficient between 0.7 and 0.8 represents acceptable internal consistency; and a coefficient below 0.7 indicates poor internal consistency, meaning the scale fails to pass the reliability test (41). The internal consistency of the questionnaire was assessed by testing the reliability of each dimension of the scale separately, with the test results presented in Table 3. As shown in the table, both the dimension-specific and overall Cronbach’s Alpha coefficients of the scale are above 0.7, demonstrating a high level of internal consistency and confirming that the questionnaire is suitable for the present study.

**Table 3:** Results of reliability analysis of the questionnaire.

Variables	Number of items	Cronbach *α*	Overall Cronbach *α*
PU	3	0.860	0.897
TU	3	0.840	
DS	3	0.814	
IS	4	0.877	
PEC	3	0.823	
MDLP	5	0.937	
A	3	0.844	
PHID	4	0.871	
MPD	3	0.853	

#### 3.3.2 Validity analysis

##### 3.3.2.1 Exploratory factor analysis (EFA)

Exploratory Factor Analysis (EFA) was conducted to test the validity of the scale, thereby verifying whether the measurement variables corresponding to each latent variable have stable consistency and a clear structural framework. Prior to the implementation of EFA, two prerequisite conditions must be satisfied (42): first, the Kaiser-Meyer-Olkin (KMO) measure of sampling adequacy should be greater than 0.7; second, the significance level of Bartlett’s Test of Sphericity should be less than 0.05. As shown in Table 4, the KMO value of the scale is 0.887 (exceeding the minimum acceptable threshold of 0.6), and the *p*-value of Bartlett’s Test of Sphericity is 0.000. These results indicate that the design of the observed variables in the questionnaire meets the prerequisite requirements for EFA, and the scale is thus suitable for exploratory factor analysis.

**Table 4:** KMO and Bartlett’s test of sphericity results.

Indicators	Value
KMO value	0.887
χ^²^	11403.385
*df*	465
*P* value	0

Subsequently, principal component analysis with an eigenvalue cutoff of 1 and varimax rotation was employed to extract common factors for further validity testing of the scale (see Table 5). The results showed that a total of 9 common factors were extracted through factor analysis, all with eigenvalues greater than 1, which are completely consistent with the 9 preset research dimensions including perceived usefulness, perceived technological usefulness, and disease severity. The cumulative variance explained by these 9 factors remains at 76.470% both before and after rotation. Rotation only alters the internal structure of the factors and the distribution ratio of variance, without changing the total explanatory power of the extracted factors. This cumulative variance explanation rate is significantly higher than the standard threshold of 60% for EFA, indicating that the 9 extracted factors can explain 76.47% of the information variation in the scale items. This proves that the factor extraction has sufficient representativeness, and the design of the scale items can effectively reflect the core connotation of the preset research dimensions.

**Table 5:** Total variance explained.

Factor Number	Characteristic root			Variance explained before rotation			Variance explained after rotation		
	Eigenvalue	Variance explained %	Cumulative%	Eigenvalue	Variance explained %	Cumulative%	Eigenvalue	Variance explained %	Cumulative %
1	8.428	27.188	27.188	8.428	27.188	27.188	4.196	13.535	13.535
2	3.441	11.099	38.288	3.441	11.099	38.288	2.965	9.566	23.101
3	2.75	8.871	47.159	2.75	8.871	47.159	2.955	9.531	32.632
4	2.357	7.604	54.763	2.357	7.604	54.763	2.38	7.677	40.309
5	1.654	5.335	60.097	1.654	5.335	60.097	2.275	7.339	47.647
6	1.549	4.996	65.093	1.549	4.996	65.093	2.274	7.334	54.982
7	1.397	4.505	69.599	1.397	4.505	69.599	2.245	7.242	62.223
8	1.117	3.603	73.202	1.117	3.603	73.202	2.242	7.233	69.456
9	1.013	3.268	76.47	1.013	3.268	76.47	2.174	7.013	76.47
10	0.562	1.813	78.282						
11	0.534	1.721	80.003						
12	0.498	1.605	81.608						
13	0.451	1.454	83.063						
14	0.436	1.406	84.469						
15	0.399	1.286	85.755						
16	0.394	1.271	87.026						
17	0.349	1.125	88.151						
18	0.343	1.106	89.257						
19	0.326	1.051	90.309						
20	0.321	1.035	91.343						
21	0.303	0.978	92.322						
22	0.297	0.958	93.279						
23	0.283	0.912	94.192						
24	0.278	0.897	95.089						
25	0.259	0.837	95.925						
26	0.258	0.832	96.757						
27	0.243	0.783	97.54						
28	0.231	0.746	98.286						
29	0.208	0.671	98.957						
30	0.175	0.566	99.523						
31	0.148	0.477	100						

Table 6 presents the rotated factor loading matrix of the EFA, which is used to clarify the corresponding relationship between scale items and latent factors and verify the construct validity of the scale. The results indicated that the absolute values of the factor loading coefficients for all items are above 0.4, indicating a strong association between the items and their corresponding latent variables. This study adopts 0.4 as the cutoff threshold for determining effective factor loadings, which is the most commonly used moderate standard in SEM research within the social sciences (43). Among them, item IS2 in the information sensitivity dimension has the highest loading coefficient (0.885), and the loading coefficients of items MDLP2 and MDLP3 in the legal protection of public health and medical data dimension both reach above 0.87, demonstrating a strong correlation between the items and their corresponding latent factors. Meanwhile, all items exhibit clear single-factor attribution with no cross-high loading phenomenon (i.e., each item only has a high loading on one specific factor and extremely low loadings on other factors). This confirms that the division of each dimension of the scale follows a rigorous logical framework with clear boundaries, and there is no confusion of dimensions or mismatch of items. In summary, the results in Table 6 verify that the scale has good construct validity, laying a solid foundation for subsequent correlation analysis and Structural Equation Model testing.

**Table 6:** Rotated factor loading coefficients.

Name	Factor 1	Factor 2	Factor 3	Factor 4	Factor 5	Factor 6	Factor 7	Factor 8	Factor 9
PU1	0.193	0.001	0.046	0.119	0.132	0.831	0.133	0.107	0.036
PU2	0.182	−0.066	0.073	0.103	0.107	0.79	0.137	0.11	0.034
PU3	0.173	0.028	0.109	0.17	−0.015	0.811	0.14	0.13	0.149
TU1	0.108	0.05	0.067	0.861	0.004	0.148	0.014	0.149	0.02
TU2	0.071	0.053	0.098	0.878	−0.001	0.133	0.028	0.134	0.028
TU3	0.126	0.036	0.018	0.818	0.151	0.075	0.055	0.116	0.072
DS1	0.056	−0.06	0.089	0.144	0.109	0.124	0.183	0.759	0.012
DS2	0.028	−0.044	0.019	0.108	0.086	0.096	0.132	0.847	0.129
DS3	0.062	0.043	0.144	0.158	0.058	0.103	0.115	0.826	0.076
IS1	−0.009	0.846	0.027	0.083	−0.043	0.013	−0.11	−0.019	0.043
IS2	0.037	0.885	0.014	−0.014	−0.02	−0.011	−0.089	0.024	−0.034
IS3	0.06	0.867	−0.03	0.04	−0.055	−0.003	−0.013	−0.026	0.053
IS4	−0.013	0.806	0.022	0.023	0.04	−0.034	−0.058	−0.037	−0.019
PEC1	0.138	−0.025	0.07	0.069	0.802	0.06	0.098	0.081	0.154
PEC2	0.122	−0.009	0.22	0.078	0.834	0.007	0.099	0.063	0.041
PEC3	0.11	−0.046	0.212	0.008	0.806	0.159	0.074	0.113	0.051
MDLP1	0.803	0.031	0.173	0.098	0.131	0.11	0.057	0.017	0.135
MDLP2	0.886	0.017	0.126	0.088	0.065	0.132	0.011	0.067	0.115
MDLP3	0.874	0.02	0.121	0.074	0.077	0.148	0.013	0.025	0.191
MDLP4	0.805	0.027	0.157	0.027	0.085	0.146	0.042	0.024	0.189
MDLP5	0.884	−0.004	0.108	0.097	0.092	0.082	0.018	0.056	0.168
A1	0.074	−0.15	0.245	0.039	0.072	0.173	0.764	0.207	0.079
A2	0.035	−0.096	0.194	0.065	0.082	0.122	0.805	0.17	0.132
A3	0.007	−0.083	0.126	0.007	0.132	0.132	0.845	0.108	0.024
PHID1	0.263	−0.009	0.777	0.076	0.099	0.108	0.085	0.009	0.208
PHID2	0.165	0.021	0.761	0.106	0.105	0.11	0.123	0.08	0.204
PHID3	0.108	0.025	0.779	0.03	0.227	0.02	0.199	0.135	0.08
PHID4	0.151	0.009	0.8	0.015	0.147	0.025	0.182	0.072	0.146
MPD1	0.272	−0.004	0.274	0.039	0.031	0.098	0.041	0.095	0.768
MPD2	0.26	0.026	0.206	0.086	0.17	0.019	0.139	0.088	0.77
MPD3	0.32	0.032	0.18	0.019	0.101	0.12	0.075	0.081	0.797

Rotation Method: Varimax (Maximum Variance) Rotation.

##### 3.3.2.2 Confirmatory factor analysis (CFA)

The results of Confirmatory Factor Analysis (CFA) are presented in Table 7. The key model fit indices selected for evaluation include the chi-square to degrees of freedom ratio (χ^2^/df), Adjusted Goodness of Fit Index (AGFI), Goodness of Fit Index (GFI), Tucker-Lewis Index (TLI), Normed Fit Index (NFI), Comparative Fit Index (CFI), and Root Mean Square Error of Approximation (RMSEA). This study follows the model fit evaluation criteria proposed by Hu and Bentler (44): a *χ*^2^/df ratio between 1 and 3 and RMSEA < 0.08 indicate acceptable fit; CFI/TLI > 0.90 and AGFI/GFI > 0.80 indicate good fit. All indices in this study meet the above standards (*χ*^2^/df = 1.836, AGFI = 0.908, GFI = 0.927, TLI = 0.965, IFI = 0.970, CFI = 0.970, RMSEA = 0.037). The same applies below.

**Table 7:** Model fit indices.

Fit index	Evaluation criterion	Statistical value	Model fit assessment
*χ*^2^/df	Ratio of Chi-square to degrees of freedom; smaller values indicate better fit. Ideally between 1 and 3.	1.836	Pass
AGFI	Comparative Fit Index; ranges 0–1, closer to 1 indicates better fit. ≥0.9 is excellent, >0.8 is acceptable.	0.908	Pass
GFI	Tucker-Lewis Index; similar to CFI, ranges 0–1, closer to 1 indicates better fit. ≥0.9 is excellent, >0.8 is acceptable.	0.927	Pass
TLI	Incremental Fit Index; ranges 0–1, closer to 1 indicates better fit. ≥0.9 is excellent, >0.8 is acceptable.	0.965	Pass
IFI	Normed Fit Index; ranges 0–1, closer to 1 indicates better fit. ≥0.9 is excellent, >0.8 is acceptable.	0.970	Pass
CFI	Relative Fit Index; ranges 0–1, closer to 1 indicates better fit. ≥0.9 is excellent, >0.8 is acceptable.	0.970	Pass
RMSEA	Root Mean Square Error of Approximation; <0.08 is acceptable, ≤0.05 is excellent.	0.037	Pass

## 4 Research results

### 4.1 Correlation analysis results

This study employs Pearson product–moment correlation analysis to examine the preliminary associations among the core variables and to detect potential multicollinearity issues. The judgment threshold refers to the approach of Tabachnick and Fidell (45) (using a correlation coefficient > 0.8 as the cutoff for identifying multicollinearity). Pearson product–moment correlation analysis was conducted to examine the interrelationships among nine core variables—perceived usefulness, perceived technological usefulness, disease severity, information sensitivity, sharing attitude, perceived behavioral control, legal protection cognition of public health and medical data, sharing willingness, and supportive attitude towards public health medical data platform construction (see Table 8). The significance level was set at *α* = 0.05, and the results are presented as follows:

**Table 8:** Correlation analysis.

	PU	TU	DS	IS	A	PEC	MDLP	PHID	MPD
PU	1								
TU	0.334***	1							
DS	0.329***	0.341***	1						
IS	−0.026	0.086*	−0.042	1					
A	0.371***	0.149***	0.399***	−0.199***	1				
PEC	0.248***	0.175***	0.258***	−0.054	0.298***	1			
MDLP	0.388***	0.244***	0.171***	0.04	0.162***	0.301***	1		
PHID	0.276***	0.201***	0.270***	0.012	0.431***	0.419***	0.407***	1	
MPD	0.297***	0.186***	0.260***	0.029	0.281***	0.314***	0.538***	0.515***	1

*p < 0.05; **p < 0.01; ***p < 0.001. The same applies below.

Most of the core variables exhibited significant correlations, with the direction of correlations generally consistent with theoretical expectations. No multicollinearity issues were detected (all correlation coefficients < 0.8), which provides a robust statistical foundation for the subsequent construction of the structural equation model (SEM) and hypothesis testing of path coefficients.

- Perceived usefulness was significantly and positively correlated with perceived technological usefulness (*r* = 0.334, *p* < 0.001), disease severity (*r* = 0.329, *p* < 0.001), sharing attitude (*r* = 0.371, *p* < 0.001), perceived behavioral control (*r* = 0.248, *p* < 0.001), legal protection cognition of public health and medical data (*r* = 0.388, *p* < 0.001), sharing willingness (*r* = 0.276, *p* < 0.001), and supportive attitude towards platform construction (*r* = 0.297, *p* < 0.001), but showed no significant correlation with information sensitivity (*r* = −0.026, *p* > 0.05). These results indicate that the improvement of perceived usefulness may positively influence technological cognition, health-related attitudes, and data sharing-related willingness, while having no obvious association with individuals’ cognition of data sensitivity levels.
- Perceived technological usefulness had a significant positive correlation with disease severity (*r* = 0.341, *p* < 0.001), information sensitivity (*r* = 0.086, *p* < 0.05), sharing attitude (*r* = 0.149, *p* < 0.001), perceived behavioral control (*r* = 0.175, *p* < 0.001), legal protection cognition of public health and medical data (*r* = 0.244, *p* < 0.001), sharing willingness (*r* = 0.201, *p* < 0.001), and supportive attitude towards platform construction (*r* = 0.186, *p* < 0.001). This suggests that individuals’ cognitive evaluation of the usefulness of big data-enabled healthcare technologies exerts a positive driving effect on multi-dimensional variables, with a relatively prominent correlation intensity with disease severity.
- Disease severity was significantly and positively correlated with sharing attitude (*r* = 0.399, *p* < 0.001), perceived behavioral control (*r* = 0.258, *p* < 0.001), legal protection cognition of public health and medical data (*r* = 0.171, *p* < 0.001), sharing willingness (*r* = 0.270, *p* < 0.001), and supportive attitude towards platform construction (*r* = 0.260, *p* < 0.001), yet no significant correlation was found with information sensitivity (*r* = −0.042, *p* > 0.05). The findings reveal that individuals’ judgment of their own health status positively affects their data sharing attitude, perceived behavioral control, and related willingness, while being independent of their cognition of data sensitivity levels.
- Information sensitivity was only significantly and negatively correlated with sharing attitude (*r* = −0.199, *p* < 0.001), and showed no significant correlations with perceived behavioral control (*r* = −0.054, *p* > 0.05), legal protection cognition of public health and medical data (*r* = 0.040, *p* > 0.05), sharing willingness (*r* = 0.012, *p* > 0.05), or supportive attitude towards platform construction (*r* = 0.029, *p* > 0.05). This indicates that individuals’ cognition of data sensitivity levels only exerts a negative effect on their sharing attitude, with no obvious impact on other core variables in the theoretical framework.
- Sharing attitude had a significant positive correlation with perceived behavioral control (*r* = 0.298, *p* < 0.001), legal protection cognition of public health and medical data (*r* = 0.162, *p* < 0.001), sharing willingness (*r* = 0.431, *p* < 0.001), and supportive attitude towards platform construction (*r* = 0.281, *p* < 0.001), with the largest correlation coefficient observed with sharing willingness. This highlights that a positive sharing attitude is the core correlated factor for enhancing individuals’ data sharing willingness, which aligns with the core logic of the Theory of Planned Behavior (TPB).
- Perceived behavioral control was significantly and positively correlated with legal protection cognition of public health and medical data (*r* = 0.301, *p* < 0.001), sharing willingness (*r* = 0.419, *p* < 0.001), and supportive attitude towards platform construction (*r* = 0.314, *p* < 0.001). The results suggest that individuals’ sense of control over the data sharing process and their confidence in risk response positively influence their cognition of legal protection mechanisms and related behavioral willingness in the context of public health data sharing.
- Legal protection cognition of public health and medical data exhibited a significant positive correlation with sharing willingness (*r* = 0.407, *p* < 0.001) and supportive attitude towards platform construction (*r* = 0.538, *p* < 0.001), with the strongest correlation intensity found with the latter. This demonstrates that a comprehensive and in-depth cognition of legal protection mechanisms for public health medical data has the most prominent impact on individuals’ supportive attitude towards the construction of public health medical data platforms, reflecting the core role of institutional trust in promoting platform construction.
- Sharing willingness was significantly and positively correlated with supportive attitude towards public health medical data platform construction (*r* = 0.515, *p* < 0.001). This finding confirms that the stronger individuals’ willingness to share personal public health data, the more positive their supportive attitude towards the construction of specialized public health medical data platforms, which verifies the intrinsic logical connection between individual behavioral willingness and institutional platform construction in the research framework.

### 4.2 Structural equation modeling and path analysis

#### 4.2.1 Model construction

Based on the Technology Acceptance Model (TAM), the Theory of Planned Behavior (TPB), and the 11 research hypotheses proposed in this study, a Structural Equation Model (SEM) was constructed to elucidate the formation mechanism of individuals’ willingness to share personal public health data in the context of big data-enabled healthcare. In this model, exogenous variables were defined as perceived technological usefulness, perceived usefulness, disease severity, and information sensitivity, which serve to explain individuals’ cognitive evaluations and objective health status related to public health data sharing. Mediating variables included sharing attitude, legal protection cognition of public health and medical data, and perceived behavioral control, which function as the critical link connecting exogenous cognitive variables and endogenous behavioral variables by transmitting the indirect effects of cognitive factors on behavioral willingness. The core endogenous variable was specified as sharing willingness, which represents the direct behavioral intention of individuals regarding public health data sharing, and the ultimate endogenous variable was supportive attitude towards public health medical data platform construction. This variable setting forms a complete action chain of cognition → psychological perception → behavioral willingness → institutional outcome in the theoretical framework, which not only integrates the core constructs of TAM and TPB but also incorporates context-specific variables (disease severity, information sensitivity, legal protection cognition) and the ultimate research outcome (platform construction support), thus reflecting the contextual characteristics of big data-enabled healthcare and the practical orientation of public health data governance.

#### 4.2.2 Model fit assessment

The maximum likelihood estimation (MLE) method was employed to estimate the model parameters, and the overall fit of the SEM was evaluated using a comprehensive set of fit indices, consistent with the evaluation criteria adopted in the confirmatory factor analysis (CFA) of the research scale to ensure the consistency of statistical testing standards. The selected fit indices included the chi-square to degrees of freedom ratio (χ^2^/df), Adjusted Goodness of Fit Index (AGFI), Goodness of Fit Index (GFI), Tucker-Lewis Index (TLI), Normed Fit Index (NFI), Comparative Fit Index (CFI), and Root Mean Square Error of Approximation (RMSEA). The recommended evaluation criteria and the actual test results of each index are presented in Table 9.

**Table 9:** Structural equation modeling fit indices.

Fit index	Evaluation criterion	Statistical value	Model fit assessment
*χ*^2^/df	Ratio of Chi-square to degrees of freedom; smaller values indicate better fit. Ideally between 1 and 3.	2.153	Pass
AGFI	Comparative Fit Index; ranges 0–1, closer to 1 indicates better fit. ≥0.9 is excellent, >0.8 is acceptable.	0.896	Pass
GFI	Tucker-Lewis Index; similar to CFI, ranges 0–1, closer to 1 indicates better fit. ≥0.9 is excellent, >0.8 is acceptable.	0.912	Pass
TLI	Incremental Fit Index; ranges 0–1, closer to 1 indicates better fit. ≥0.9 is excellent, >0.8 is acceptable.	0.952	Pass
NFI	Normed Fit Index; ranges 0–1, closer to 1 indicates better fit. ≥0.9 is excellent, >0.8 is acceptable.	0.922	Pass
CFI	Relative Fit Index; ranges 0–1, closer to 1 indicates better fit. ≥0.9 is excellent, >0.8 is acceptable.	0.957	Pass
RMSEA	Root Mean Square Error of Approximation; <0.08 is acceptable, ≤0.05 is excellent.	0.043	Pass

The results in Table 9 show that the χ^2^/df value of the model is 2.153, which falls within the reasonable range of 1 to 3, indicating a good overall fit of the structural model and no over-fitting problem. The AGFI and GFI values are 0.896 and 0.912, respectively, both exceeding the critical threshold of 0.8, which reflects a strong explanatory power of the theoretical model for the empirical data and a high degree of consistency between the model structure and the data distribution. The TLI, NFI, and CFI values are 0.952, 0.922, and 0.957, respectively, all above the ideal threshold of 0.9, which further verifies that the theoretical structure of the model is highly consistent with the actual distribution of the sample data and that the model has excellent incremental fit. The RMSEA value is 0.043, which is less than the acceptable range of 0.08 and close to the excellent level of 0.05, indicating a small approximate error of the model and a good parsimonious fit. Collectively, all fit indices of the SEM constructed in this study meet the recommended statistical standards, confirming the good adaptability of the model. On this basis, further path relationship testing and research hypothesis verification can be conducted to explore the direct and indirect effects among variables.

#### 4.2.3 Path testing results

Path analysis was conducted to test the significance and effect intensity of the preset causal relationships among all variables in the SEM. The validity of each hypothetical path was judged based on four statistical indicators: standardized path coefficient (*β*), standard error (SE), critical ratio (CR), and *p*-value (46). The statistical testing criteria were set as follows: an absolute CR value > 1.96 indicates a significant path relationship with *p* < 0.05; an absolute CR value > 2.58 indicates a highly significant path relationship with *p* < 0.01; an absolute CR value > 3.29 indicates an extremely significant path relationship with *p* < 0.001. The standardized path coefficients, CR values, and *p*-values of all preset paths are presented in Table 10.

**Table 10:** Path coefficients and hypothesis testing results.

Path			Path coefficient	SE	CR	*p*
PU	←	TU	0.407	0.057	8.582	***
A	←	PU	0.311	0.038	7.043	***
MDLP	←	PU	0.437	0.044	9.703	***
A	←	DS	0.364	0.037	7.963	***
PEC	←	IS	−0.062	0.044	−1.333	Not significant
A	←	IS	−0.211	0.046	−5.000	***
PHID	←	A	0.379	0.042	8.729	***
PHID	←	MDLP	0.312	0.035	7.677	***
PHID	←	PEC	0.308	0.048	7.248	***
MPD	←	PHID	0.404	0.049	9.062	***
MPD	←	MDLP	0.421	0.04	9.800	***

The results of the path test show that 10 out of the 11 preset hypothetical paths passed the significance test, with only the path of information sensitivity → perceived behavioral control failing to reach the significant level (*β* = −0.062, *p* = 0.182), meaning Hypothesis H5 proposed in this study is not supported by the empirical data. The specific analysis of the significant path relationships is as follows:

Perceived technological usefulness exerts an extremely significant positive effect on perceived usefulness (*β* = 0.407, *p* < 0.001), which verifies Hypothesis H1. This result indicates that individuals’ cognitive evaluation of the practical utility of big data-enabled healthcare technologies can effectively enhance their recognition of the value and usefulness of public health data sharing, reflecting the core explanatory power of TAM in the context of healthcare data sharing.

Perceived usefulness has an extremely significant positive impact on sharing attitude (*β* = 0.311, *p* < 0.001) and legal protection cognition of public health and medical data (*β* = 0.437, *p* < 0.001), supporting Hypotheses H2 and H3. It reveals that the stronger individuals’ perception of the usefulness of public health data sharing, the more likely they are to form a positive sharing attitude, and the more they pay attention to the legal protection mechanisms for data sharing, which links the technology acceptance theory with the institutional trust cognition in the research context.

Disease severity exerts an extremely significant positive effect on sharing attitude (*β* = 0.364, *p* < 0.001), confirming Hypothesis H4. This finding suggests that individuals with a higher perception of their own disease severity have a more positive attitude towards public health data sharing, as they tend to recognize the clinical and public health value of data sharing for disease diagnosis, treatment, and prevention, which reflects the objective health status as an important contextual factor influencing individuals’ sharing attitude.

Information sensitivity has an extremely significant negative impact on sharing attitude (*β* = −0.211, *p* < 0.001), supporting Hypothesis H6, but its negative effect on perceived behavioral control is not significant (*β* = −0.062, *p* = 0.182), rejecting Hypothesis H5. This indicates that individuals’ high sensitivity to data privacy and security will only directly lead to a negative sharing attitude, but will not significantly reduce their sense of control over the data sharing process or their confidence in risk response, which implies that privacy concerns mainly act on the attitudinal level rather than the behavioral control level in the formation of sharing willingness.

Sharing attitude (*β* = 0.379, *p* < 0.001), legal protection cognition of public health and medical data (*β* = 0.312, *p* < 0.001), and perceived behavioral control (*β* = 0.308, *p* < 0.001) all exert an extremely significant positive effect on sharing willingness, with sharing attitude having the strongest effect intensity. These results verify Hypotheses H7, H8, and H9, and are highly consistent with the core logic of TPB—attitude, subjective norm (replaced by legal protection cognition in this study), and perceived behavioral control are the three key direct predictors of behavioral willingness.

Sharing willingness (*β* = 0.404, *p* < 0.001) and legal protection cognition of public health and medical data (*β* = 0.421, *p* < 0.001) both have an extremely significant positive impact on supportive attitude towards public health medical data platform construction, supporting Hypotheses H10 and H11. Notably, the effect intensity of legal protection cognition is slightly higher than that of sharing willingness, which demonstrates that a comprehensive cognition of legal protection mechanisms is the most important driving factor for individuals to support the construction of public health medical data platforms, followed by their own data sharing willingness.

The results of SEM and path analysis clearly reveal the multi-level action mechanism among variables in the theoretical framework: perceived technological usefulness indirectly acts on sharing attitude and legal protection cognition through perceived usefulness; disease severity and information sensitivity regulate sharing willingness by directly influencing sharing attitude (with the former having a positive effect and the latter a negative effect); sharing attitude, legal protection cognition, and perceived behavioral control are the direct key factors affecting sharing willingness; finally, sharing willingness and legal protection cognition jointly drive individuals’ supportive attitude towards the construction of public health data platforms. The constructed model not only verifies most of the research hypotheses with good fit but also effectively explains the formation path of individuals’ willingness to share personal public health data in the context of big data-enabled healthcare. It clarifies the direct and indirect effects, as well as the effect intensity, among cognitive factors, psychological perceptions, behavioral willingness, and institutional outcomes, thus providing an accurate and reliable theoretical basis for the formulation of subsequent practical strategies to promote public health data sharing and the construction of medical data platforms.

## 5 Discussion and limitations

### 5.1 Core research findings

This study examines the factors influencing the willingness of the Chinese population to share health data, and its findings are consistent with those of the global systematic review conducted by Atalay and Yücel (47): perceived usefulness of technology, legal protection, and perceived behavioral control all serve as core drivers of health data sharing on a global scale. Notably, the Chinese case exhibits a unique characteristic: legal protection exerts a slightly stronger impact on platform construction than on individuals’ willingness to share data. This phenomenon is closely associated with China’s current policy context, where the development of medical big data is promoted through “institutional construction.”

#### 5.1.1 The driving effect of perceived technological usefulness and perceived usefulness

Perceived technological usefulness exerts a significant positive impact on perceived usefulness, indicating that individuals’ cognition of the innovativeness and practicality of big data-enabled healthcare technologies can effectively enhance their recognition of the value of data sharing. When users perceive that big data-enabled healthcare technologies have high practical value and can bring tangible benefits to health management, they are more inclined to regard data sharing as useful. This result is consistent with the core viewpoint of the Technology Acceptance Model, verifying the important impact of technological characteristics on users’ cognition. Meanwhile, perceived usefulness further exerts a positive impact on sharing attitude and cognition of legal protection, which means that if users perceive that data sharing can meet their health management needs, they are more likely to form a positive sharing attitude and pay greater attention to relevant legal safeguard measures. This also provides an important entry point for improving users’ willingness to share data in subsequent practices.

#### 5.1.2 The moderating effect of disease severity and information sensitivity

Disease severity has a positive impact on sharing attitude. Users with poor health status have a higher recognition of the value of data sharing for disease prevention and control, thus holding a more positive sharing attitude. This phenomenon conforms to realistic logic: groups with poor health status usually have higher demands for medical services and are more eager to obtain precise health assessments and medical advice through data sharing, hence having a higher acceptance of data sharing. Information sensitivity exerts a negative impact on sharing attitude but has no significant effect on perceived behavioral control, indicating that users’ concerns about the level of data sensitivity indirectly affect their willingness to share data mainly through attitude, rather than directly influencing their perception of control over data. This implies that users’ concerns about data privacy will lead them to form a negative sharing attitude, but will not affect their cognition of their own ability to control the data sharing process. Therefore, in the process of improving users’ willingness to share data, it is particularly important to alleviate their concerns about data privacy and guide the formation of a positive sharing attitude.

#### 5.1.3 The key role of attitude, legal protection and perceived behavioral control

Sharing attitude, legal protection of public health and medical data, and perceived behavioral control all significantly and positively predict the willingness to share data, among which attitude has the largest impact coefficient, indicating that a positive sharing attitude is the core factor for improving the willingness to share. This result confirms the viewpoint of the Theory of Planned Behavior: as individuals’ subjective evaluation of behavior, attitude directly affects the formation of behavioral willingness. The improvement of the legal protection mechanism for public health and medical data can alleviate users’ privacy concerns and enhance their trust in data sharing, thereby improving their willingness to share data. Perceived behavioral control can strengthen users’ confidence in responding to the risks of data sharing; when users believe that they can control the data sharing process and cope with potential risks, they are more willing to participate in data sharing. These three factors jointly constitute the core factors influencing the willingness to share data, providing a clear direction for formulating strategies to improve the willingness to share data.

#### 5.1.4 The promoting effect of sharing willingness and legal protection on platform construction

Both the willingness to share data and the legal protection of public health and medical data exert a significant positive impact on the construction of public health data platforms, indicating that users’ positive willingness to participate and sound legal safeguards are important supports for promoting the large-scale construction and operation of data platforms. The improvement of users’ willingness to share data can provide rich data resources for the platform and promote the optimization and improvement of platform functions. A sound legal protection mechanism can enhance users’ trust in the platform, attract more users to participate in platform construction and data sharing, and form a positive cycle. This result highlights the importance of users’ willingness and institutional guarantees in the construction of public health data platforms, providing an important reference for the construction and promotion of such platforms.

#### 5.1.5 Analysis of the reasons for the unsupported hypothesis

Hypothesis H3 (the positive impact of perceived usefulness on the legal protection of public health and medical data is not significant) failed to pass the test, and the possible reasons are as follows: First, users’ perception of the value of data sharing and their cognition of legal safeguards belong to different dimensions. Perceived usefulness focuses on technological and behavioral value, while cognition of legal protection focuses on institutional security, leading to a weak correlation between the two. Second, the current publicity and popularization of laws and regulations on public health data protection in China are insufficient. Some users have a low level of understanding of relevant legal systems, and even if they recognize the practical value of data sharing, it is difficult for them to form a clear cognition of legal safeguards. Third, some users have concerns about the implementation effect of the legal system, believing that existing laws cannot effectively avoid the risk of privacy leakage, which results in the insignificant impact of perceived usefulness on the cognition of legal protection.

### 5.2 Practical implications

#### 5.2.1 Technological optimization dimension

Based on the research result that “perceived technological usefulness has a significant positive impact on perceived usefulness, and perceived usefulness further positively affects sharing attitude,” the usability and practicality of big data-enabled healthcare platforms should be improved to enhance users’ perceived technological usefulness and perceived usefulness of data sharing. Specifically, simplify the data upload process and optimize the operation interface of the platform to lower the operational threshold for users, and highlight the core functions closely related to users’ personal health management (such as precise health assessment, personalized health advice and chronic disease monitoring) to make users clearly perceive the practical value of data sharing and big data technology. At the same time, based on the research conclusion that “information sensitivity negatively affects sharing attitude,” the platform should clearly display the data security protection measures on the prominent interface (such as data access authority control, data encryption processing and privacy protection rules), so as to alleviate users’ concerns about data privacy leakage and reduce the negative impact of information sensitivity on sharing attitude.

#### 5.2.2 Legal safeguard dimension

Improve the laws and regulations related to the protection of public health and medical data, clarify the boundaries of data sharing, privacy protection standards and responsibility division, and establish a data security traceability mechanism and a liability investigation system for privacy leakage. Strengthen the publicity and popularization of legal policies, raise users’ awareness of and trust in the legal system for data protection, help users understand the legal safeguards for data sharing, and boost their confidence in participating in data sharing. Specifically, it is necessary to further refine the legal boundaries of health data sharing based on the requirements of the Personal Information Protection Law (PIPL): clarify the scope of data collection for medical institutions and big data platforms, establish a hierarchical protection system for sensitive health information, improve the civil compensation and administrative accountability mechanisms after privacy leakage, and enhance the public’s trust in the implementation effect of PIPL.

#### 5.2.3 Publicity and guidance dimension

Carry out targeted publicity for users with different health status and information sensitivity levels, and emphasize the dual value of data sharing for personal health management and public health governance. Eliminate users’ misunderstandings and concerns about data sharing through case explanations, popular science publicity and other methods, and guide the formation of a positive sharing attitude. Meanwhile, build a user communication platform, encourage users to share their experiences and gains from data sharing, give play to the demonstration effect, and improve the public’s acceptance of data sharing.

#### 5.2.4 Platform construction dimension

Construct a unified public health and medical data platform, integrate multi-source medical data resources, and realize interconnection and efficient utilization of data. Smooth user feedback channels, timely understand users’ needs and concerns, and continuously optimize platform functions and services. Establish a user participation mechanism, encourage users to participate in platform construction and management, enhance users’ sense of identity and belonging to the platform, and strengthen their sense of control and participation in data sharing.

### 5.3 Research limitations and future prospects

The Chinese experience derived from this study provides a reference for global medical big data governance: when promoting health data sharing in developing countries, it is necessary to balance technological optimization and institutional guarantee, and in particular, strengthen the public’s awareness of legal protection to alleviate privacy concerns. Although this study has achieved certain results, it still has the following limitations: First, the most prominent limitation of this study is the youth/technological literacy bias in the sample: 81.17% of the participants were young people aged 19–39, and there was a complete absence of older population participants aged 60 and above. However, the older population are the core demanders of medical services, and the factors influencing their willingness to share health data may differ significantly from those of young people (e.g., lower perceived usefulness of technology and weaker awareness of legal protection). This bias prevents the conclusions of this study from being generalized to the entire age group, especially the older population and groups with low digital literacy. Second, the study focuses on cross-sectional data and cannot reveal the dynamic interaction between variables. Future research can adopt longitudinal tracking studies to explore the long-term change trends of variable relationships. Third, the measurement of legal protection only focuses on the user cognitive level, without involving objective dimensions such as the effect of legal implementation and the degree of institutional improvement, so the measurement dimensions need to be enriched.

Based on the above limitations, future research can be carried out in the following directions: First, expand the sample scope, adopt stratified sampling, increase the proportion of samples from older population groups, low-education groups, rural groups and other populations, and improve the generalizability of research conclusions. Second, conduct longitudinal tracking studies to dynamically analyze the interaction between variables and reveal the formation and change mechanism of individuals’ willingness to share personal public health data. Third, combine the application of emerging technologies (such as blockchain and artificial intelligence) in data privacy protection to explore the synergistic mechanism of technological empowerment and institutional guarantee, so as to provide more targeted suggestions for the construction of a public health data sharing system.

## Data Availability

The original contributions presented in the study are included in the article/supplementary material, further inquiries can be directed to the corresponding author.
